# Antibiotic resistance in Swiss nursing homes: analysis of National Surveillance Data over an 11-year period between 2007 and 2017

**DOI:** 10.1186/s13756-018-0378-1

**Published:** 2018-07-20

**Authors:** Philipp Kohler, Rosamaria Fulchini, Werner C. Albrich, Adrian Egli, Carlo Balmelli, Stephan Harbarth, Delphine Héquet, Christian R. Kahlert, Stefan P. Kuster, Christiane Petignat, Matthias Schlegel, Andreas Kronenberg

**Affiliations:** 1 Division of Infectious Diseases and Hospital Epidemiology, Kantonsspital St. Gallen, St. Gallen, Switzerland; 2 Clinical Microbiology Division, University Hospital Basel, Basel, Switzerland; 3 Applied Microbiology Research, Department of Biomedicine, University of Basel, Basel, Switzerland; 4 Servizio di prevenzione delle infezioni e medicina del personale, Ente Ospedaliero Cantonale, Ticino, Switzerland; 5 Division of Infectious Diseases and Infection Control Program, Geneva University Hospitals and Faculty of Medicine, Geneva, Switzerland; 6 Unité cantonale hygiène, prévention et contrôle de l’infection, Canton of Vaud, Switzerland; 7 Division of Infectious Diseases and Hospital Epidemiology, Children’s Hospital of Eastern Switzerland, St. Gallen, Switzerland; 8 Division of Infectious Diseases and Hospital Epidemiology, University and University Hospital Zurich, Zurich, Switzerland; 9 Institute for Infectious Diseases, University Bern, Bern, Switzerland; 10 Swiss Centre for Antibiotic resistance (ANRESIS), Bern, Switzerland

**Keywords:** Antibiotic resistance, Long-term care facility, Nursing homes, Extended-spectrum cephalosporin resistance, Methicillin-resistant *Staphylococcus aureus*, Switzerland

## Abstract

**Background:**

We evaluated data from isolates of nursing home (NH) patients sent to the Swiss centre for antibiotic resistance (ANRESIS). We focussed on carbapenem-resistance (CR) among Gram-negative pathogens, extended-spectrum cephalosporin-resistant (ESC-R) *Escherichia coli*/*Klebsiella pneumoniae*, methicillin-resistant *Staphylococcus aureus* (MRSA), and glycopeptide-resistant enterococci (GRE).

**Methods:**

NH patient isolates from 01/2007 to 10/2017 were extracted. Temporal trends in resistance were described and risk factors associated with ESC-R and MRSA were assessed. For every administrative subdivision in Switzerland (i.e. canton), we calculated a coverage rate, defined as number of beds of governmentally-supported nursing homes, which sent ≥1 isolate in each 2014, 2015, and 2016, divided by the total number of supported beds.

**Results:**

We identified 16′804 samples from 9′940 patients. A majority of samples (12′040; 71.6%) originated from the French/Italian speaking part of Switzerland. ESC-R *E. coli* increased from 5% (16/299) in 2007 to 22% (191/884) in 2017 (*P* < 0.01), whereas MRSA decreased from 34% (35/102) to 26% (21/81) (*P* < 0.01). Provenience from the German (vs. French/Italian) speaking part of Switzerland was associated with decreased risk for ESC-R (OR 0.5, 95% CI 0.4–0.7) and for MRSA (OR 0.1, 95% CI 0.1–0.2). CR among *Pseudomonas aeruginosa* was 10% (105/1096) and showed an increasing trend over time; CR among *Enterobacteriaceae* (37/12′423, 0.3%) and GRE (5/1′273, 0.4%) were uncommon. Overall coverage rate was 9% (range 0–58% per canton). There was a significant difference between the French/Italian (median 13%, interquartile range [IQR] 4–43%) and the German speaking cantons (median 0%, IQR 0–5%) (*P* = 0.02).

**Conclusions:**

ESC-R among *E. coli* is emerging in Swiss NHs, whereas MRSA show a declining trend over time. A minority of NHs are represented in ANRESIS, with a preponderance of institutions from the French/Italian speaking regions. Efforts should be undertaken to improve resistance surveillance in this high-risk setting.

**Electronic supplementary material:**

The online version of this article (10.1186/s13756-018-0378-1) contains supplementary material, which is available to authorized users.

## Background

Long-term care facilities (LTCF) have been recognized as an important reservoir for antibiotic resistant pathogens [1–3]. In a recent point-prevalence study from four different LTCF in Italy, the prevalence of extended-spectrum beta-lactamase (ESBL)-carriage was 57% and of methicillin-resistant *Staphylococcus aureus* (MRSA) 17% [4]. Also in Italy, the prevalence of carbapenemase-producing *Enterobacteriaceae* (CPE) - one of the most concerning antibiotic resistance threats - ranged from 1 to 6.3% [5]. Residence in an LTCF has been shown to be a risk factor for carriage of MRSA and glycopeptide-resistant enterococci (GRE) [6, 7]. Risk factors for resistant pathogens among nursing home (NH) residents include previous antibiotic treatment, invasive devices, age, open wounds, sharing a room with a colonized patient, bedriddenness, or high degree of disability [2, 4, 5, 8, 9].

In Switzerland, studies on antibiotic resistance from NHs or other LTCFs have mostly focussed on MRSA and have almost exclusively been generated in the Western part of the country [10–13]. Previously published national surveillance data on MRSA and extended-spectrum cephalosporin resistant (ESC-R) *Enterobacteriaceae* from Switzerland have not included data from LTCF [14, 15]. In light of the alarming trends in antibiotic resistance from LTCF in neighboring countries, we aimed to assess temporal trends in the prevalence of antibiotic resistant isolates from patients in Swiss NHs and to identify risk factors associated with resistance.

## Methods

### Setting

This observational laboratory-based study was conducted using the database from the Swiss antimicrobial resistance surveillance network (ANRESIS) available since 2004 [16]. This database provides antibiotic resistance data for all routinely collected microbiological samples from 20 clinical microbiology laboratories, distributed all over Switzerland and representing at least 70% of annual hospitalization days and 30% of all Swiss general practitioners. For this analysis, we included all isolates sent to ANRESIS from Swiss NHs.

Antimicrobial susceptibility testing was performed at local laboratories according to Clinical and Laboratory Standards Institute (CLSI) or European Committee on Antimicrobial Susceptibility Testing (EUCAST) guidelines [17, 18]. Most of the participating laboratories switched from CLSI to EUCAST breakpoints between 2011 and 2013. All laboratories are participating in at least one external quality program out of the National External Quality Assessment Service or the Swiss quality control program from the Institute for Medical Microbiology, University of Zurich [19, 20].

### Inclusion criteria and definitions

We extracted resistance data for all *Enterobacteriaceae*, *Pseudomonas aeruginosa*, *Acinetobacter* spp., *Staphylococcus aureus *and *Enterococcus faecium/faecalis *which were sent to ANRESIS between January 2007 until October 2017 with the patient label “long-term care” and which originated from Swiss NHs. These pathogens represent 81% of NH isolates sent to ANRESIS during this time period. Although data were anonymous, every patient possesses a unique identity number within their institution, which was used to exclude repeat isolates (i.e. same pathogen and resistance profile) from the same patient in a specific calendar year. Each of the 26 administrative subdivisions in Switzerland (i.e. cantons) was assigned to either the French/Italian (cantons of Fribourg, Geneva, Jura, Ticino, Valais and Vaud), or the German speaking part (all other cantons). Based on the name of the institution linked to the isolate we distinguished the following types of institutions: i) governmentally supported NHs according to publicly available lists issued by the Federal Office of Public Health (FOPH) [21]; and ii) not governmentally supported NHs.

For *Enterobacteriaceae*, *Pseudomonas aeruginosa *and *Acinetobacter* spp., carbapenem-resistance (CR) was studied. For *Escherichia coli* and *Klebsiella pneumoniae*, ESC-R as well as co-resistances to quinolones, aminoglycosides, nitrofurantoin, and fosfomycin were evaluated. Oxacillin- or cefoxitin-resistance among *Staphylococcus aureus* was used as a proxy for methicillin-resistant *S. aureus* (MRSA). Glycopeptide-resistant *Enterococcus faecalis/faecium* (GRE) were also studied. Resistance to a particular substance was defined as either intermediate or resistant susceptibility test according to the individual laboratories. Group resistance (e.g. glycopeptide, carbapenem, aminoglycoside or quinolone resistance) was defined as resistance or intermediate susceptibility to at least one antibiotic of the respective group, ESC-R was defined as resistance or intermediate susceptibility to at least one antibiotic tested out of 3rd or 4th generation cephalosporins.

### Calculation of coverage rate

For every canton, we estimated the coverage rate of governmentally supported NHs sending microbiology isolates to ANRESIS. This was achieved by dividing the number of beds represented by institutions in the dataset who sent at least one isolate to ANRESIS in 2014, 2015, and 2016 by the total number of beds. The number of supported beds was collected from publicly available lists of the FOPH for the year 2016 [21, 22].

### Data analysis and statistics

Categorical variables were reported as frequencies and proportions, continuous variables as median with interquartile range (IQR). For dichotomous variables, Chi-square or Fisher-exact tests were used, as appropriate. For comparison of coverage rates between geographic regions, the Mann-Whitney-U was used. For each type of resistance, the proportion of pathogens with the resistance trait (as % of the total number of isolates reported and tested for the respective key antibiotic) was analysed over time. Linear regression analysis was used to estimate the association between resistance (independent variable) and year of surveillance (dependent variable).

For ESC-R and MRSA, univariable and multivariable logistic regression analysis with generalized estimating equation (GEE) were performed to assess factors independently associated with resistance. The following co-variables were assessed: Patient age group (i.e. < 70, 70–85, > 85 years), sex, geographical region (i.e. French/Italian, German), and site of sample collection (i.e. urogenital, skin, respiratory, other). Co-variables were entered into multivariable analysis in case of a *P* value< 0.1 in univariable analysis. Two-sided *P* values ≤0.05 were considered statistically significant. SAS Studio was used for all statistical analyses.

### Sensitivity analysis

To eliminate potential selection bias, a sensitivity analysis was performed assessing resistance time trends exclusively for NHs which sent microbiological isolates every year between 2007 and 2017 (only for ESC-R and MRSA, as the sample sizes for the other pathogens were too small).

## Results

### Data sources

After removal of duplicates, we identified 19′189 tested pathogens from 16′804 samples and 9′940 patients. Of the samples, 14′663 (87.3%) were from governmentally funded NHs and 2′141 (12.7%) from non-funded NHs. A majority of samples (12′040; 71.6%) originated from the French/Italian and 4′764 (28.4%) from the German speaking part of Switzerland.

### ESC-R for ***E. Coli*** and *K. pneumoniae*

**Fig. 1:**
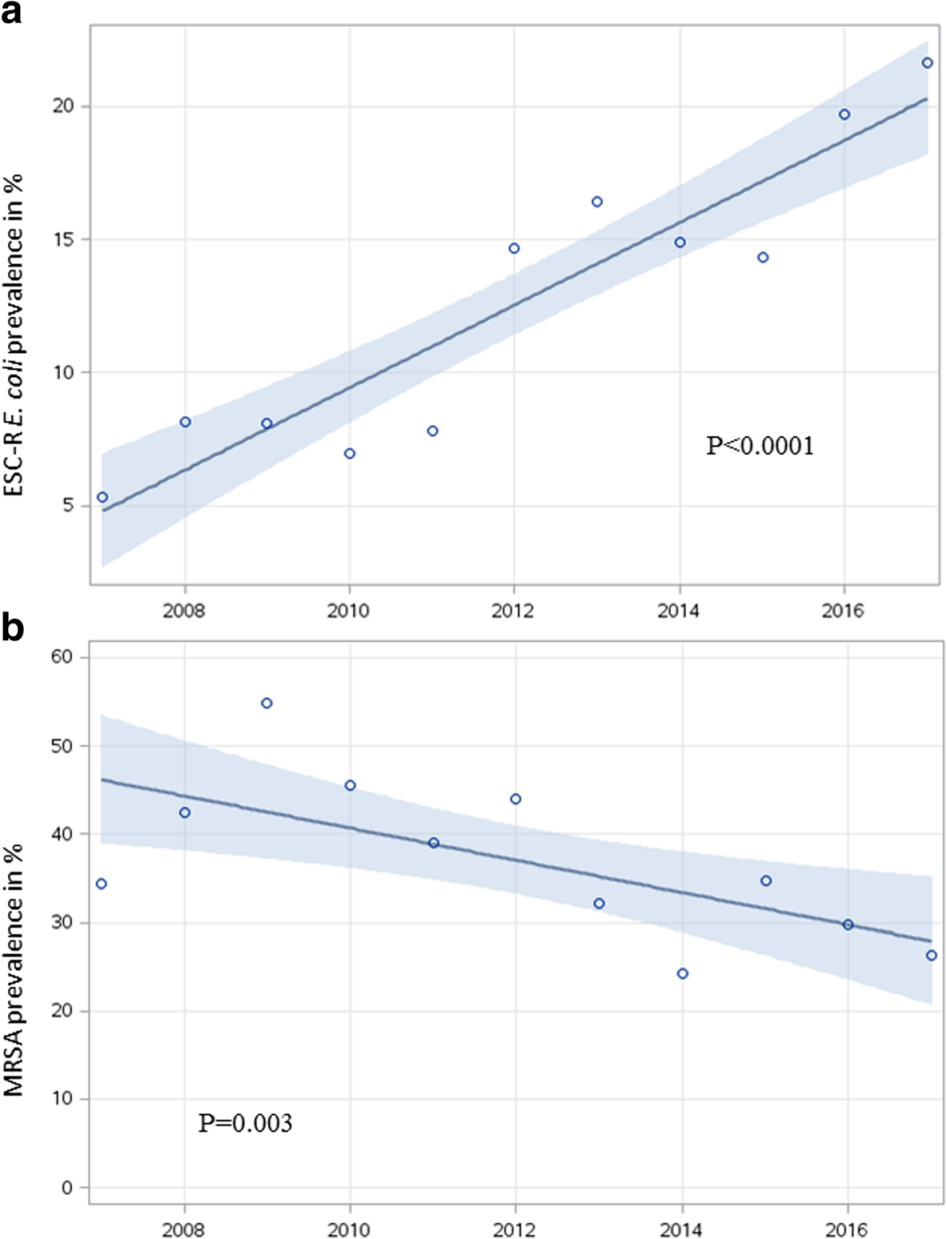
Resistance trends (in %, y-axis) of ESC-R *Escherichia coli* (**a**) and of methicillin-resistant *Staphylococcus aureus* (**b**) in Swiss nursing homes between 2007 and 2017 using a linear regression model (including 95% confidence interval in light blue)

**Fig. 2:**
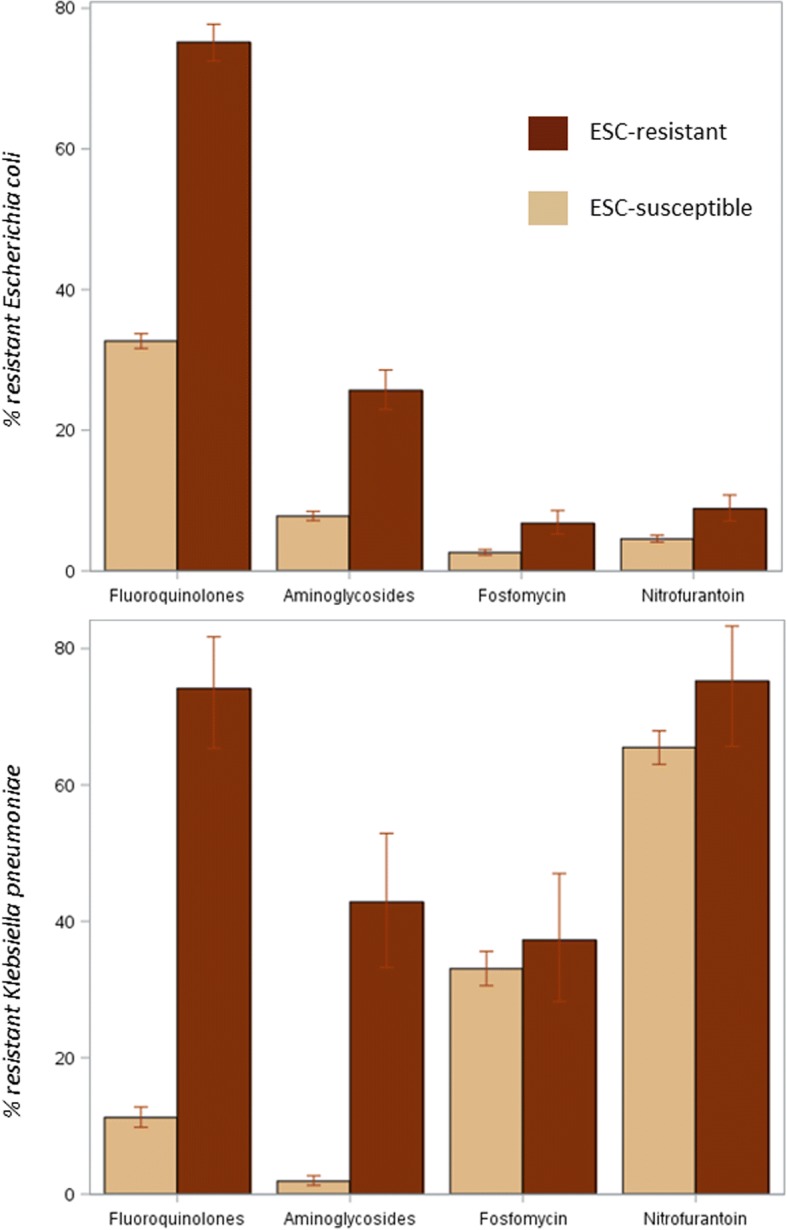
Co-resistances of *Escherichia coli *and *Klebsiella pneumoniae* isolates from Swiss nursing home residents between 2007 and 2016, by resistance to extended-spectrum cephalosporins (ESC), including 95% confidence intervals

We identified 8′607 *E. coli* and 1′825 *K. pneumoniae* isolates, mostly from the urinary tract (97% of *E. coli* and 94% of *K. pneumoniae*). ESC-R was more common among *E. coli* (14.1%) compared to *K. pneumoniae* (7.0%). From 2007 to 2017, ESC-R *E. coli* increased from 5.4% (16/299) to 21.6% (191/884) (*P* < 0.0001) (Fig. 1a). A similar but less pronounced increase from 1.6 to 7.8% was observed for ESC-R *K. pneumoniae* (*P* < 0.0001). In multivariable analysis, isolates originating from the German speaking part of Switzerland (vs. French/Italian; OR 0.5, 95% CI 0.4–0.7) were less likely, and those from men (vs. women; OR 1.6, 95% CI 1.2–2.1) more likely to exhibit ESC-R (Table 1). Co-resistances were more frequent in the ESC-R group, for both *E. coli* and *K. pneumoniae*. For ESC-R *E. coli*, resistance to fluoroquinolones was 75%, whereas resistance to aminoglycosides, nitrofurantoin and fosfomycin were 25.7, 8.8 and 6.8%, respectively (Fig. 2).

**Table 1:** Patient characteristics and univariable and multivariable logistic regression using generalized estimating equation for evaluation of risk factors for ESC-R in patients with *Escherichia coli* and *Klebsiella pneumoniae* isolates from Swiss NHs between 2007 and 2016

	ESC-S	ESC-R		Univariable		Multivariable	
	*n* = 9′093	*n* = 1′339	Row %^a^	OR (95% CI)	*P* value	OR (95% CI)	*P* value
Sex					**0.03**		**0.003**
Female	7′436	1′063	12.5	ref.		ref.	
Male	1′657	276	14.3	1.4 (1.0–1.9)		1.6 (1.2–2.1)	
Age in years							
< 70	713	110	13.4	ref.		–	
70–85	5′479	799	12.7	0.9 (0.7–1.2)	0.60		
> 85	2′901	430	12.9	1.0 (0.8–1.2)	0.74		
Region							
French/Italian speaking	6′407	1′037	13.9	ref.		ref.	
German speaking	2′686	302	10.1	0.5 (0.4–0.7)	**<.0001**	0.5 (0.4–0.7)	**<.0001**
Site							
Urogenital	8′788	1′286	12.8	ref.		–	
Other	305	53	14.8	1.4 (0.7–2.5)	0.34		
Species							
*E. coli*	7′396	1′211	14.1	ref.		ref.	
*K. pneumoniae*	1′697	128	7.0	0.2 (0.1–0.3)	**<.0001**	0.2 (0.1–0.3)	**<.0001**

*Abbreviations: ESC-R* Extended-spectrum beta-lactam resistant, *ESC-S* Extended-spectrum beta-lactam susceptible, *NH* Nursing Home, *OR* Odds Ratio, *CI* Confidence Interval, *ref* Reference

Significant *P* values are in bold and non-significant ones (*P* ≥ 0.05) are not in bold

^a^ESC-R/(ESC-S + ESC-R)

### Carbapenem-resistance (CR)

Among *Enterobacteriaceae*, 37 of 12′423 tested isolates (0.3%) were resistant to carbapenems. CR was more common among patients less than 70 years of age (*P* = 0.01) and those from the French/Italian speaking regions (*P* = 0.01); no time trend was observed (*P* = 0.14). Among *P. aeruginosa*, 105 of 1′096 isolates (9.6%) showed CR, with an increasing trend from 2007 (2%) to 2016 (8%) (*P* = 0.0001). Non-urogenital isolates (thereof 68% skin and 25% respiratory isolates) more frequently exhibited CR (12.3%) than urogenital samples (8.4%, *P* = 0.04). Among 83 tested *Acinetobacter* spp. isolates, none exhibited CR. Additional file 1: Table S1 gives an overview on the isolates included in this analysis.

### Methicillin-resistant *Staphylococcus aureus*

Among 1′482 isolates of *S. aureus*, 556 (37.5%) were resistant to methicillin. Prevalence decreased over time from 34.3% (35/102) in 2007 to 25.9% (21/81) in 2017 (*P* = 0.004) (Fig. 1a). Geographical provenance was the only factor associated with methicillin-resistance in multivariable analysis, with isolates from the German part being less commonly resistant than those from French/Italian speaking parts of Switzerland (OR 0.1, 95% CI 0.1–0.2, *P* < 0.0001) (Table 2).

**Table 2:** Patient characteristics and results of univariable and multivariable logistic regression using generalized estimating equation for risk factors for methicillin-resistance in patients with *Staphylococcus aureus* isolates from Swiss nursing homes between 2007 and 2017

	MSSA	MRSA		Univariable		Multivariable	
	*n* = 926	*n* = 556	Row %^a^	OR (95% CI)	*P* value	OR (95% CI)	*P* value
Sex							
Female	542	298	35.5	ref.		ref.	
Male	384	258	40.2	1.4 (0.9–2.2)	0.11	1.6 (1.0–2.5)	0.06
Age in years							
< 70	133	57	30.0	ref.		ref.	
70–85	563	361	39.1	1.5 (1.1–2.2)	**0.02**	1.3 (0.9–1.9)	0.13
> 85	230	138	37.5	1.5 (1.0–2.2)	**0.04**	1.3 (0.8–1.9)	0.27
Region							
French/Italian speaking	697	498	41.7	ref.		ref.	
German speaking	229	58	20.2	0.1 (0.1–0.2)	**< 0.0001**	0.1 (0.1–0.2)	**< 0.0001**
Sampling site							
Urogenital	259	174	40.2	ref.		ref.	
Skin	492	294	37.4	0.9 (0.7–1.2)	0.44	0.9 (0.7–1.1)	0.20
Other	175	88	33.5	0.8 (0.5–1.1)	0.09	0.7 (0.5–1.0)	0.06

*Abbreviations: MSSA* Methicillin-susceptible *Staphylococcus aureus*, *MRSA* Methicillin-resistant *Staphylococcus aureus*, *OR* Odds Ratio, *CI* Confidence Interval

Significant *P* values are in bold and non-significant ones (*P* ≥ 0.05) are not in bold

^a^ESC-R/(ESC-S + ESC-R)

### Glycopeptide-resistant enterococci

GR among enterococci was uncommon (5/1′273; 0.4%). Due to the small number of resistant isolates, no further analyses were performed. Additional file 1: Table S1 gives an overview on the isolates included in this analysis.

### Sensitivity analysis

Considering only institutions participating in ANRESIS from 2007 throughout to 2017, the same time trends could be observed for ESC-R (15 institutions, 1′555 isolates) with resistance increasing from 4.2% in 2007 to 21.7% in 2017 (*P* < 0.0001). For MRSA (14 institutions, 201 isolates), the high proportion of resistance persisted from 2007 (42.4%) to 2017 (60%) (*P* = 0.61).

### Coverage of governmentally supported institutions

Considering 11′584 samples from governmentally funded NHs which sent samples throughout 2014 to 2016, the national coverage rate of supported NH beds was 9%. Whereas some cantons did not provide any isolates to ANRESIS, others had coverage rates of up to 58%, again with a significant difference between the French/Italian (median 13%, IQR 4–43%) and the German (median 0%, IQR 0–5%) speaking part of the country (*P* = 0.02). Additional file 1: Figure S1 shows the coverage rate for every canton.

## Discussion

In this analysis of national resistance data from Swiss NHs, we show that ESC-R have been clearly increasing between 2007 and 2017 reaching 22% among *E. coli* isolates, whereas the proportion of MRSA among *S. aureus* isolates is declining. The nationwide collection of NH isolates and the inclusion of data over more than a decade are notable strengths of this study and increase its validity and significance.

The analysis of the available resistance data shows a significant increase of ESC-R among *E. coli* and *K. pneumoniae*. These findings mirror the increase of ESC-R observed in an ANRESIS analysis of inpatient and outpatient isolates from Swiss acute care institutions [15]. According to the ANRESIS database, the proportion of ESC-R among invasive *E. coli* acute care isolates for the year 2016 was 9%, compared to 18% in our data [23]. This supports the notion of NHs being a high-risk setting for ESBL-producing pathogens. In contrast to the study by Kronenberg et al., where isolates from the German part of Switzerland exhibited slightly more frequently ESC-R, we found - on the contrary - a significantly higher resistance rate among isolates originating from French/Italian speaking regions [15]. Indeed, particularly for resistant Gram-negative pathogens, the countries bordering Switzerland in the South (Italy) and in the West (France) have reported a high ESBL prevalence of 58% among NH residents and 28% among patients on geriatric wards, respectively [4, 24]. In Germany and Austria, somewhat lower ESBL carriage rates of 18 and 13% have been reported from these settings [25, 26]. In our study, male sex was clearly associated with ESC-R, which has also been described for patients in Switzerland not residing in NHs [15]. Although several other studies have documented an association of male sex with carriage of or infection with ESBL-producing organisms [27–29], others found female sex to be associated with ESBL-carriage or infection [30–32]. It has been suggested that these conflicting findings might be due to differences in antibiotic prescribing practices for women with uncomplicated cystitis [27]. Interestingly, the proportion of ESC-R *E. coli* with co-resistance to fluoroquinolones was high at 75% in our study. Whether this high proportion is due to the dissemination of the frequently fluroroquinolone-resistant ST131 *E. coli* clone in Swiss NHs (as shown for other countries) should be further evaluated [33–35]. Based on our resistance data and in accordance with national antibiotic treatment guidelines, nitrofurantoin and fosfomycin remain reasonable options for the empirical treatment of NH patients, at least for uncomplicated urinary tract infections [36].

We found the proportion of MRSA among *S. aureus* isolates to be declining between 2007 and 2017. This is in line with national acute care resistance data, but also with two point-prevalence studies performed in 2010/11 and 2015 among residents of NH in the canton of Vaud [10, 13, 14]. In addition, samples from the German speaking part of Switzerland less commonly exhibited methicillin-resistance than those from French/Italian speaking parts, which also has been shown for the Swiss acute care setting [14]. Nevertheless, these data - and particularly the high resistance rates of over 50% in 2009 - should be interpreted with caution because i) the absolute number of *S. aureus* isolates was relatively small (*N* = 1′482), and ii) the decreasing trend over time could not be confirmed in the sensitivity analysis.

CR among *P. aeruginosa* from NH residents is increasing in all geographic regions in Switzerland. Potential explanations include the increasing use of carbapenems and/or the change from CLSI to EUCAST breakpoints, which has been shown to lead to decreased susceptibility rates, in particular for *P. aeruginosa* tested for carbapenems [37–39]. The proportion of CR among *Enterobacteriaceae* and *Acinetobacter* spp. as well as glycopeptide resistance among enterococci is negligible in Swiss NHs. Nevertheless, especially for CR among *Enterobacteriaceae*, regular surveillance is indicated in light of their emergence in geographically close regions such as Northern Italy, where LTCF are known high-risk settings for these pathogens.

Only 9% of governmentally-supported NH beds in Switzerland are represented in ANRESIS and from many cantons in the German speaking part of Switzerland, LTCF isolates are lacking completely. We can only speculate on the reasons for these gaps in the reporting system. One potential reason is that NH residents are often treated by their family physicians, and therefore the samples are not attributed to the institution, but to general physicians. By only including samples which could be unambiguously ascribed to NHs, we also decreased the number of samples and the statistical power of our analysis. However, the credibility and validity of our results are strengthened through this approach.

Our study has several limitations. First, the overrepresentation of the French/Italian speaking part of the country hampers the generalizability of our analysis. Still, we believe that the available data are of interest to public health authorities, clinical microbiologists, hospital epidemiologists, and also to clinicians in care of NH patients. Second, comparability of laboratory data across different institutions over more than a decade can be debated. As mentioned above, several laboratories have switched from CLSI to EUCAST breakpoints during the observed time period. Also, we cannot exclude inconsistencies in testing for particular antibiotic substances such as fosfomycin, which requires the determination of the minimal inhibitory concentration in the presence of glucose-6-phosphate. Third, we cannot fully exclude the possibility that collection practices changed during the observed time period or between geographical regions in Switzerland, which might have confounded our results. Fourth, it remains debatable to what extent ESC-R can be used as a proxy for ESBL-production. However, in the 2016 antibiotic resistance report of the European Centre for Disease Prevention and Control, 89% of ESC-R *E. coli* were ESBL-producers [40]. Fifth, our approach of calculating a coverage rate for every canton can be discussed. Because the number of samples sent per institution was not considered in the calculation, large institutions sending few samples to ANRESIS will be overrepresented compared to small institutions sending many samples. Sixth, because certain pathogens such as streptococci are not included in our analysis, the coverage rate might be slightly underestimated. However, we found that the isolates in our study represent 81% of all NH isolates, which allows to reasonably estimate the coverage rate.

## Conclusions

The prevalence of ESC-R *E. coli* and *K. pneumoniae* among Swiss NHs has clearly been increasing over the last decade. In this analysis of mostly urinary samples, nitrofurantoin and fosfomycin retained high susceptibility rates against *E. coli*, even for ESC-R isolates. The proportion of MRSA among *S. aureus* seems to be declining. Efforts should be undertaken to increase the coverage of NHs samples in the national resistance database ANRESIS, especially in the German speaking parts of Switzerland.

## Additional file

Additional file 1: **Table S1.** Number of isolates included in the analysis by pathogen vs. sex, age group, site of detection and geographical region. **Figure S1.** Total and in ANRESIS represented proportion (i.e. coverage rate) of governmentally supported nursing home beds per canton. (DOCX 101 kb)

## Abbreviations

ANRESIS: Swiss antimicrobial resistance surveillance network
CLSI: Clinical and laboratory standards institute
CPE: Carbapenemase-producing Enterobacteriacae
CR: Carbapenem-resistance
ECDC: European centre for disease prevention and control
ESBL: Extended-spectrum β-lactamase
ESC-R: Extended-spectrum cephalosporin resistant
EUCAST: European committee on antimicrobial susceptibility testing
FOPH: Federal office of public health
GEE: Generalized estimating equation
GRE: Glycopeptide-resistant enterococci
IQR: Interquartile range
LTCF: Long-term care facility
MRSA: Methicillin-resistant *Staphylococcus aureus*
NH: Nursing home
OR: Odds ratio
