# Supplementary material for: Antibiotic resistance in Swiss nursing homes: analysis of National Surveillance Data over an 11-year period between 2007 and 2017
Source: Antimicrob Resist Infect Control. 2018 Jul 20;7:88. doi: 10.1186/s13756-018-0378-1 (PMC6053768; doi:10.1186/s13756-018-0378-1)

**Additional file 1**

**Table S1**. Number of isolates included in the analysis by pathogen *vs.* sex, age group, site of detection and geographical region.

|  | **Total** | **Sex** | **Age** | | | **Site** | | | | **Region** | |
| --- | --- | --- | --- | --- | --- | --- | --- | --- | --- | --- | --- |
|  |  | Fem | <70 | 70-85 | >85 | Uro | Skin | Resp | Other | F/I | G |
| Enterobacteriaceae^a^ | 12423 | 9678 | 1067 | 7440 | 3916 | 11587 | 620 | 112 | 104 | 9217 | 3206 |
| *Escherichia coli* | 7783 | 6486 | 534 | 4664 | 2585 | 7542 | 180 | 21 | 40 | 5784 | 1999 |
| *Klebsiella* spp. | 1993 | 1493 | 211 | 1193 | 589 | 1862 | 86 | 32 | 13 | 1416 | 577 |
| *Proteus* spp. | 1395 | 899 | 152 | 847 | 396 | 1145 | 214 | 18 | 18 | 1142 | 253 |
| *Enterobacter* spp. | 442 | 275 | 59 | 264 | 119 | 364 | 43 | 22 | 13 | 301 | 141 |
| *Citrobacter* spp. | 392 | 257 | 45 | 231 | 116 | 349 | 28 | 4 | 11 | 256 | 136 |
| Other | 418 | 268 | 66 | 241 | 111 | 325 | 69 | 15 | 9 | 318 | 100 |
| *Pseudomonas aeruginosa* | 1096 | 591 | 182 | 645 | 269 | 762 | 228 | 83 | 23 | 707 | 389 |
| *Acinetobacter* spp. | 83 | 40 | 15 | 50 | 18 | 56 | 15 | 7 | 5 | 54 | 29 |
| *Staphylococccus aureus* | 1482 | 840 | 190 | 924 | 368 | 433 | 786 | 146 | 117 | 1195 | 287 |
| *Enterococcus faecalis* | 1143 | 669 | 150 | 699 | 294 | 1004 | 121 | 1 | 17 | 984 | 159 |
| *Enterococcus faecium* | 130 | 98 | 7 | 83 | 40 | 123 | 3 | 0 | 4 | 90 | 40 |
| Abbreviations: Fem, Female; Uro, Urogenital; Resp, Respiratory; F/I, French and Italian speaking part; G, German speaking part | | | | | | | | | | | |
| ^a^ Number of isolates analysed for carbapenem-resistance. Please note that for analysis of resistance towards extended-spectrum cephalosporines, the number of *E. coli* and *K. pneumoniae* isolates tested was higher than reported in this table (see Table 1 in the manuscript). | | | | | | | | | | | |

**Figure S1**. Total and in ANRESIS represented proportion (i.e. coverage rate) of governmentally supported nursing home beds per canton.


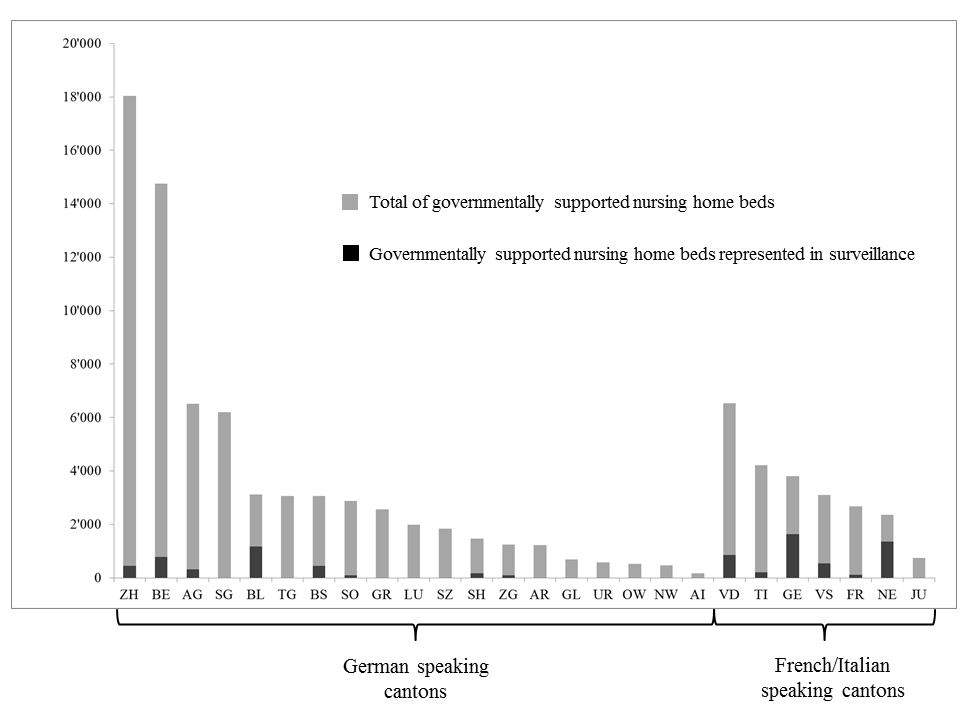

Supplement: Supplementary file 1 — Table S1. Number of isolates included in the analysis by pathogen vs. sex, age group, site of detection and geographical region. Figure S1. Total and in ANRESIS represented proportion (i.e. coverage rate) of governmentally supported nursing home beds per canton. (DOCX 101 kb) [file 13756_2018_378_MOESM1_ESM.docx]
